# Pollen-Mediated Movement of Herbicide Resistance Genes in *Lolium rigidum*

**DOI:** 10.1371/journal.pone.0157892

**Published:** 2016-06-23

**Authors:** Iñigo Loureiro, María-Concepción Escorial, María-Cristina Chueca

**Affiliations:** Plant Protection Department, Weed Control Group, Instituto Nacional de Investigación y Tecnología Agraria y Alimentaria (INIA), Madrid. Spain; California State University, Fresno, CA, UNITED STATES

## Abstract

The transfer of herbicide resistance genes by pollen is a major concern in cross-pollinated species such as annual ryegrass (*Lolium rigidum*). A two-year study was conducted in the greenhouse, under favorable conditions for pollination, to generate information on potential maximum cross-pollination. This maximum cross-pollination rate was 56.1%. A three-year field trial was also conducted to study the cross-pollination rates in terms of distance and orientation to an herbicide-resistant pollen source. Under field conditions, cross-pollination rates varied from 5.5% to 11.6% in plants adjacent to the pollen source and decreased with increasing distances (1.5 to 8.9% at 15 m distance and up to 4.1% at 25 m in the downwind direction). Environmental conditions influenced the cross-pollination both under greenhouse and field conditions. Data were fit to an exponential decay model to predict gene flow at increasing distances. This model predicted an average gene flow of 7.1% when the pollen donor and recipient plants were at 0 m distance from each other. Pollen-mediated gene flow declined by 50% at 16.7 m from the pollen source, yet under downwind conditions gene flow of 5.2% was predicted at 25 m, the farthest distance studied. Knowledge of cross-pollination rates will be useful for assessing the spread of herbicide resistance genes in *L*. *rigidum* and in developing appropriate strategies for its mitigation.

## Introduction

A native of the Mediterranean region, *Lolium rigidum* Gaud. (annual or rigid ryegrass) is the most important ryegrass species in Spain where it is commonly found as a major weed in winter cereal crops. It is also widespread across other grain-growing regions in Southern Europe, in Southern and Western Australia, in different states of the USA, and in some areas of South Africa, Chile and Argentina. For years, it has been controlled by pre- or post-emergence herbicides. The excellent efficacy of these herbicides encouraged their widespread repeated use in several countries. This selection process led *L*. *rigidum* to become a major problem because of its ability to evolve resistance to the several herbicides used for its control [1,2]. To date, *L*. *rigidum* has evolved resistance to 11 herbicide modes of action in 12 countries [3]. Surveys conducted in Australia [4–6], USA [7] and Spain [8,9] have revealed widespread occurrence of herbicide-resistant *Lolium* species, with biotypes resistant to almost all herbicides available for its control, including biotypes with cross- and multiple-resistances. This situation complicates its management and threatens the productivity and sustainability of current cereal farming systems in these countries. The problem is even greater considering that no major herbicide with a new mode of action has been introduced to the market for more than 20 years [10].

*L*. *rigidum* is an obligate cross-pollinated species with high genetic variability [11,12]. The spread of herbicide resistance may be aided by the pollen-mediated gene flow (PMGF) among resistant and susceptible plants, which can result in an exchange and progressive accumulation of different herbicide resistance alleles present in populations [13]. Genes can move from plant to plant and among patches at short distances and longer distances to adjacent fields. Even fields with no history of herbicide use can have higher than expected initial frequencies of herbicide resistance genes due to gene flow [14]. Farmers perceive gene flow, via seed or pollen, to be a major factor in the occurrence of resistance [15]. In wind-pollinated and highly outcrossed species, as is the case of *L*. *rigidum*, gene flow by pollen is of utmost importance. In this species, resistance conferred by dominant nuclear genes will spread rapidly since the resistance alleles are not lost in the heterozygous progeny under herbicide pressure, considerably reducing the number of years necessary to evolve resistance [16]. In addition, if herbicide is applied at low doses, polygenic broad-spectrum herbicide resistance can evolve by the accumulation of quantitative genes of small effect through cross-pollination among survivors [17,18]. Despite its great importance, the contribution of PMGF to herbicide resistance dispersal is poorly understood and its importance could be underestimated. In recent times, PMGF has received considerable attention in the context of risk assessment of genetically modified (GM) crops and the possibility of transgene transfer to non-GM varieties and wild relatives [19]. In general, few PMGF studies have been conducted in wind-pollinated weedy grasses and very little data exist on *Lolium* spp. Maxwell [20], using *L*. *multiflorum* (Italian ryegrass) and diclofop-resistance as marker, found 1% cross-pollination at about 7 m, with resistant seeds collected up to 24 m. About 99% of the expected crosses occurred within 8.6 m from a 1 m^2^ pollen source. Giddings [21, 22] showed that pollen flow in *L*. *perenne* (perennial ryegrass) had a leptokurtic distribution and reached up to 80 m distance from the source. For the same species, Cunliffe et al [23] showed that gene flow declined from 20% at 0 m to < 2% at > 36 m. To our knowledge very few studies on PMGF have been conducted in *L*. *rigidum*. Only Busi et al [24] studied pollen-mediated flow of herbicide resistance genes in *L*. *rigidum* and found 37.8% of cross-pollination at 0 m from the pollen source using a single susceptible pollen receptor plant (no pollen competition), while this value was three times lower (12.9%) when two receptor plants were used. In this study, 100 m was the next distance in which cross-pollination was measured (0.93%) and cross-pollination was observed up to 3,000 m.

PMGF can be even important in autogamous species with high levels of self-compatibility as in the case of *Echinochloa crus-galli* (barnyardgrass), with cross-pollination rates up to 12.5% at 0 m distance [25]. In other grasses as *Festuca arundinacea* (tall fescue), gene flow rates of 5% were detected at 50 m and less than 1% at 150 m [26], while in *Agrostis stolonifera* (creeping bentgrass) effective gene transfer was observed at distances up to 21 km [27]. In studies conducted with dicotyledonous weeds such as *Kochia scoparia* (kochia, mock-cypress), cross-pollination rates declined from 13% at 1.5 m to 1.4% per plant at 29 m from the resistant plants [28]. In *Amaranthus palmeri* (Palmer amaranth), up to 50–60% of the offspring at 1 and 5 m were resistant to glyphosate, whereas 20–40% were resistant up to the 250 and 300 m distances [29]. For species that lack an effective seed dispersal mechanism, such as *Chenopodium album* (common lambsquarters), average cross-pollination at 2 m was 3%. PMGF plays an important role in the transfer and frequency of resistance alleles within and between populations [30].

The objective of this study was to assess *L*. *rigidum* PMGF under a medium-distance scale field experiment conducted under semi-arid conditions. PMGF was evaluated by tracking the mobility of an herbicide resistance gene from a resistant pollen source to susceptible recipient plants.

## Materials and Methods

The study was carried out at the INIA experimental station, Madrid, Spain (40° 27´ North; 3° 44´ West).

### Plant Material

A diclofop-methyl resistant *L*. *rigidum* AUS 96 biotype (R) was used as the pollen donor in the study. This biotype has an ACCase (Acetyl CoA-Carboxylase) insensitive to inhibition by APP herbicides (aryloxyphenoxypropionates: diclofop, fluazifop and haloxyfop) conferring a high degree of resistance. A single dominant nuclear gene confers ACCase target-site-based resistance in this biotype [31]. The susceptible (S) population 314, collected in a weed survey conducted in the year 2000 in Castilla-León and characterized as highly susceptible (100% mortality at diclofop-methyl field rate of 540 g a.i. ha^-1^), was used as pollen receptor.

### Potential Cross-Pollination in the Greenhouse

To establish the potential rate of cross-pollination, open pollination crosses between R and S biotypes were performed in 2007 and 2008 in the greenhouse. Plants of both biotypes were grown in a greenhouse in which lateral walls were open, and temperature and relative humidity were similar to outdoor conditions. Plants were protected from rain but without additional control of light or temperature. The pollen donor was the *L*. *rigidum* R biotype sown at a density of 500 plants m^-2^ in a 9 x 3 x 0.5 m table containing manure, sand, and soil (1:1:1 by volume). Several sets of the S biotype were sown in pots (9 plants per pot) periodically at 15-day intervals at four different times. The plants were watered as required. Two to 3 days before the anthesis of the R biotype, ten pots of the S biotype that overlapped in their flowering with the R pollinator were placed inside the pollen source 1.5 m away from each other and surrounded by R plants for their crossing. At maturity, the seeds produced by all the plants growing in each S pot were harvested, threshed, and stored for 6 months prior to the herbicide screening. Maximum and minimum temperatures, rainfall, and relative humidity data were collected during the flowering period for all experimental runs when there was a flowering overlap between pollen-donor and -receptor (Table 1).

**Table 1:** Meteorological data for the *L*. *rigidum* pollination periods by year and cross-pollination experiment (greenhouse and field experiment).

Year	Pollination period	Temperature (°C)			HR (%)	Rainfall mm	Prevailing wind	
	(n° days)					(n° days)		
		Mean	Maximum	Minimum			Direction	Avg. speed
							(n° days)	(km h^-1^)
30-year	May	16.7	22.2	11.3	53	50	--	--
						(7.3)		
Greenhouse								
2007	5–31 May	15.6 ± 2.7	22.6 ± 3.9	8.9 ± 2.6	56 ± 15	28.0	--	--
	(26)					(6)		
2008	9–31 May	13.9 ± 1.4	18.4 ± 1.4	9.0 ± 2.1	70 ± 8	28.0	--	--
	(22)					(16)		
Field plot								
2009	14–29 May	18.9 ± 2.6	26.5 ± 4.8	10.9 ± 2.6	42 ± 14	28.0	N-NE; S-SW	9.5 ± 3.4
	(16)					(3)	(7); (5)	
2010	10 May-2 June	17.6 ± 4.2	24.8 ± 5.5	11.2 ± 3.9	51 ± 9	4.6	N-NW; NE	8.4 ± 3.0
	(24)					(3)	(10); (6)	
2011	1–20 May	16.7 ± 1.8	23.3 ± 2.8	10.1 ± 2.3	63 ± 7	24.4	N-NW; N-NE	9.6 ± 4.9
	(20)					(6)	(6); (6)	

Mean and standard deviation (SD) of the maximum and minimum average temperatures, relative humidity (RH), rainfall (total mm and number of days with precipitation), and wind data (cardinal direction and speed) collected during the overlap in the flowering periods between *L*. *rigidum* pollen-donor and -receptor biotypes.

### Pollen Mediated Gene Flow under Field Conditions

The study to evaluate pollen-mediated gene flow in *L*. *rigidum* under field conditions was conducted in the 2008–09, 2009–10 and 2010–11 growing seasons. *L*. *rigidum* plants for the field experiments were obtained by vernalization of the seeds of the R and S populations in the third week of December each season. Seeds were pre-germinated in plastic trays on two layers of filter paper moistened with tap water and covered with a transparent plastic film to hold in moisture, and placed in a growth chamber set at 22⁄16 ± 1°C day⁄night for two days to initiate germination. Then the trays containing the seeds were placed in a refrigerated chamber for six weeks (S seeds) and eight weeks (R seeds) at 4°C with a photoperiod of 16 h of light (100 μE m^-2^ s^-1^ PAR, photosynthetically active radiation) and 8 h of darkness. In early February, at the 1–2 leaf stage, seedlings were transplanted to 75 L plastic containers (48 cm diameter and 42 cm height) filled with manure, sand, and soil (1:1:1 by volume) placed in the experimental plot. Two sets of S containers were sown at 15-day interval to ensure overlap in the flowering time with the R pollen source. At the time of flowering of the pollen source, the S containers that overlapped with the R were chosen and placed in the distances at each direction and those that were not synchronized were removed.

**Fig 1:**
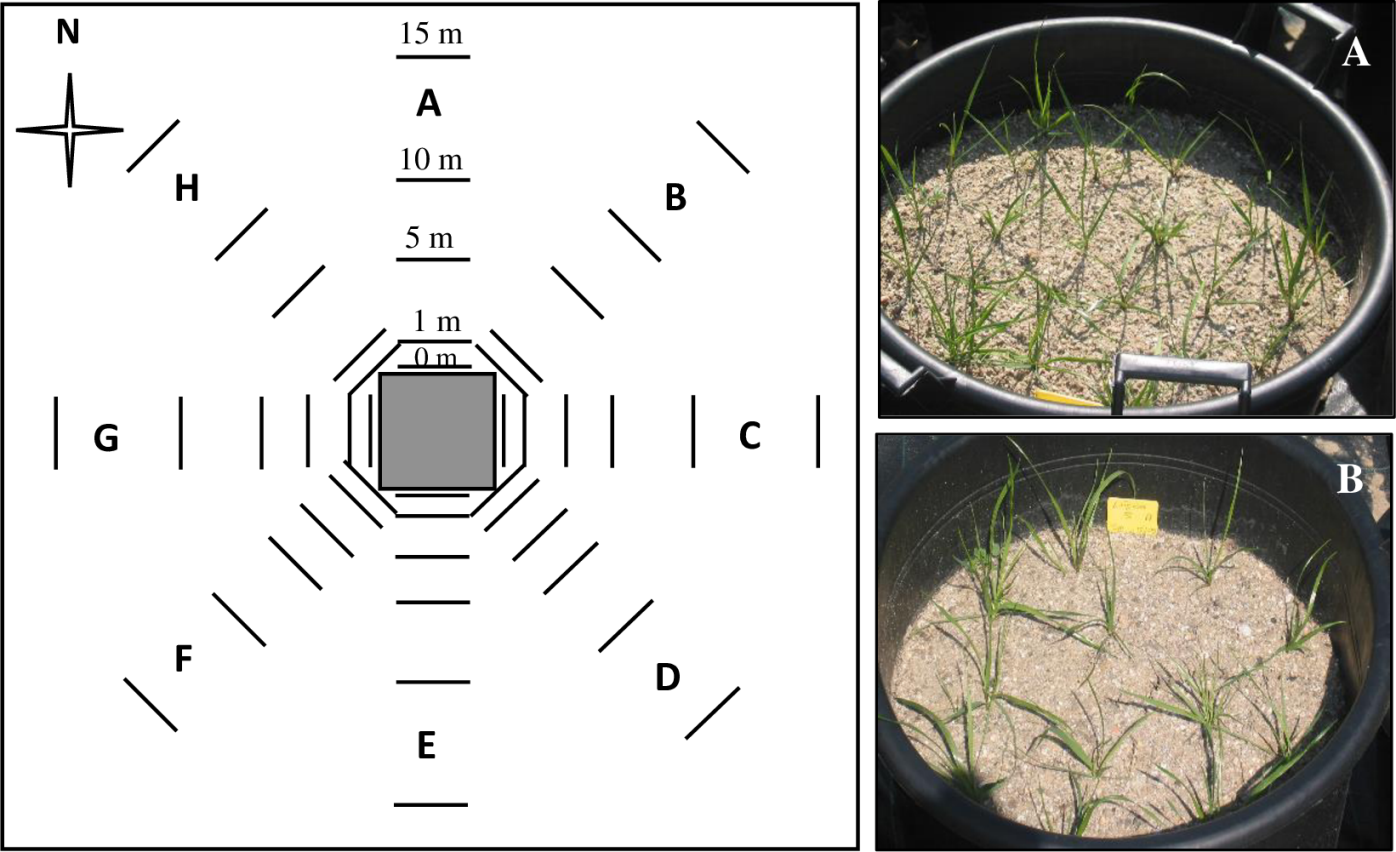
Experimental design of the pollen mediated gene flow experiment. The central pollen donor source consisted of 3 x 3 m square of *L*. *rigidum* biotype resistant (R) to diclofop-methyl (36 containers with 25 resistant ryegrass seedlings each, A), surrounded by pots placed in eight directions and at five distances (0, 1, 5, 10 and 15 m) containing plants of a susceptible (S) *L*. *rigidum* biotype (3 containers per sampling point, 12 susceptible seedlings each, B).

The field trial design was such that it permitted observations on the extent of crossing at different distances and directions from the pollen source. Each year the experimental design consisted of a 3 x 3 m central square pollen source of a diclofop resistant pollen donor at a density of 100 plants m^-2^ (36 containers with 25 resistant ryegrass seedlings each) and receptor containers placed in eight cardinal directions (A: North; B: Northeast; C: East, D: Southeast, E: South, F: Southwest, G: West and H: Northwest) of the pollen source at distances of 0, 1, 5, 10 and 15 m (Fig 1). In the wind direction (sides C and D), receptors were also placed at distances of 20 and 25 m. Three S receptors were placed at each distance in each direction, with 12 S plants per container (36 plants m^-2^). The dates of anthesis were recorded. At maturity, plants from each S container placed at different distances were individually harvested, pooled, hand threshed, and seeds were stored until herbicide screening. Harvest was made twice within a two-week interval. Meteorological data (maximum and minimum temperatures, rainfall, relative humidity, and wind velocity and direction) was collected throughout the pollen source flowering periods each year (Table 1). Data on 30-year monthly average temperatures and precipitation was obtained from the Meteorological State Agency of Spain.

### Cross-Pollination Detection by Herbicide Screening

Six months after harvest, the seeds produced in each S recipient container were screened for cross-pollination by herbicide treatment. In the case of cross-pollination under greenhouse conditions, all the seeds produced by each S plant were screened. In the field study, for each S container, 600 seeds per tray and three trays per container were screened, resulting in 5,400 seeds screened for each distance (detection threshold of 0.1% at a probability level of 99%). Seeds were sown in 22 L (40 x 30 x 18 cm) plastic trays filled with the soil mix described earlier. An additional three trays were sown with the S and R biotypes, as controls for herbicide response. The average germination was determined by counting the number of emerged seedlings per tray in relation to the amount of seeds sown. At the 3–4 leaf stage, the plants were sprayed with diclofop-methyl (Iloxan EC, 360 g a.i. L^-1^, Bayer Crop Science) at 540 g a.i. ha^-1^, the recommended field rate. Herbicide was applied using a stationary sprayer with one Teejet 8002-E flat fan nozzle delivering 225 L ha^-1^ at 200 kPa. After spraying, the trays were placed in the glasshouse and watered as required. Temperature was maintained at 24⁄16 ± 2°C (day ⁄ night). Seedlings that survived the spray were subjected to a second application of diclofop-methyl at the same rate. Survival was recorded 3 weeks later and the plants that survived the two applications of diclofop-methyl were considered as R hybrids.

### Data Analysis

Data on the cross-pollination rates were subjected to an arcsin√x transformation prior to a multi-factorial variance analysis (ANOVA). The year effects were considered as random in both greenhouse and field studies; direction and distance in relation to the pollen source were considered as fixed effects in field studies. Differences among means were tested by a Newman-Keuls test at P = 0.05. Statgraphics Centurion XVI.II software [32] was used to carry out the statistical analysis. A 95% confidence interval for the mean of cross-pollination at each distance was calculated assuming a binomial distribution. An exponential decay model used in other PMGF studies [33, 34] was fitted to the data to estimate the potential cross-pollination rate in relation with distance: *P* = *a* ∙ *e*^−*bx*^, where P is the percent of cross-pollination (%); x is the distance (m) from the pollen source; a is the cross-pollination when x = 0 and b is a non-linear coefficient determining the slope of the curve (decline rate). The curve was fitted using generalized non-linear least squares (Splus package) with error assumed to be normally distributed. The adequacy of the curve was assessed by RMSE (root mean square error) and EF (modelling efficiency) statistical indicators, which were used to assess the model goodness of fit and to compare measured and predicted values. The RMSE and EF values were calculated based on an equations: $RMSE=\sqrt{\frac{1}{n}}\sum_{i=1}^{n}{\left(Oi-Pi\right)}^{2}$ and $EF=1-\frac{\sum_{i=1}^{n}{\left(Pi-Oi\right)}^{2}}{{\sum_{i=1}^{n}(Oi-Ō)}^{2}}$, where Oi and Pi are observed and predicted values and n the total number of observations. Smaller RMSE value means better fit to the model due to closer observed and predicted values, while EF can range from -∞ to 1, with values closer to 1 meaning more accurate predictions. The distances wherein frequency of gene flow was reduced by 50% (O_50_) were estimated from the exponential decay function following the equation [35]: $O_{50=}\frac{ln0.5a-lna}{-b}$, where a is the intercept and b the slope.

## Results

### Potential Cross-Pollination in the Greenhouse

In 2007, the overlap in the flowering periods was from 5 to 31 May. During this period, average minimum and maximum temperatures were 8.9°C and 22.6°C respectively, with an average relative humidity (RH) of 56%. In 2008 (9 to 31 May), the minimum temperatures during the overlapping period were similar to those in 2007 but maximum temperatures were lower (18.4°C), and RH was higher (70%) (Table 1). The germination of the seeds was 89% and 55% in 2007 and 2008, respectively. The cross-pollination rates were significantly (P < 0.05) different between the two years of the study. A higher percentage of R hybrids was obtained in 2008 than in 2007 (44.2 ± 8.1% in 2008 compared to 14.3 ± 4.1% in 2007).

### Pollen Mediated Gene Flow under Field Conditions

The flowering between the R and S biotypes was synchronous ensuring cross-pollination in the three years of the study. The meteorological data for the experimental area during the overlap in the flowering periods and for the 30-year average (1981–2011) in Madrid are shown in Table 1. The plants flowered earlier in 2011 than in the previous years, in which the flowering periods were similar. The flowering lasted 16 days in 2009, 24 in 2010, and 20 in 2011. The 30-year average temperature in May, the month in which cross-pollination occurs under semi-arid conditions, was 16.7°C (22.2°C for the maximum and 11.3°C for the minimum temperatures). The average annual precipitation was 421 mm with 50 mm occurring in May alone, making it the month with the highest amount of rainfall. All 3 years of the study was hotter and drier than normal, especially 2009 and 2010. The maximum temperatures during the flowering period occurred in 2009 with an average of 26.5°C. In this year, a total of 28 mm of rainfall was received in 3 days at the beginning of May and the average RH was 42%, the lowest of the 3-year period. In 2010, maximum temperatures from 10 May-2 June averaged 24.8°C and the RH 51% (3 days with precipitation, 4.6 mm). The 2011 pollination period was from 1 to 20 May. This period was also warmer than the 30-year average but less than the previous years. RH in the 2011 pollination period averaged 63% (6 days with precipitation, 24 mm). The average wind speed and prevailing directions, from the N (N-NE and N-NW), were similar during the pollination periods over the 3-year period, although in 2009 wind blew from the S-SW during several days.

A total of 216,000 seeds (1,800 seeds per sampling point x 8 directions x 5 distances x 3 years) from recipient plants were screened in the greenhouse for diclofop-methyl resistance. The germination rates ranged from 83% to 91% in the three years. Table 2 (S1 Appendix) the PMGF rates recorded in different directions and distances and the average pollen mediated gene flow rates by distance obtained in the three years in which the assay was performed. The ANOVA conducted showed differences between years (F_2,340_ = 18.1, P = 0.001) and distances (F_4,340_ = 6.0, P = 0.01). Year by distance interaction was also significant (F_8,340_ = 12.0, P < 0.001), assessing the variation among pollination conditions and winds from different years. Wind direction affected (F_7,340_ = 3.8, P = 0.01) the percentage of cross-pollination, that was greatest downwind (sides E, C, D and G since wind direction was from the N, Table 1, Fig 1). The year by direction interaction was not significant (F_14,340_ = 0.9, P = 0.49).

**Table 2:** Pollen-mediated gene flow between diclofop-methyl resistant and susceptible *Lolium rigidum* biotypes at five distances (0, 1, 5, 10 and 15 m) to eight directions from the pollen source in a field experiment conducted in 2009, 2010, and 2011 at the INIA experimental station in Madrid, Spain.

Distance (m)	Pollen-mediated gene flow (%)									95% CI	
	A	B	C	D	E	F	G	H	Avg.	Lower	Upper
**2009**											
0	2.3	5.9	6.7	5.5	8.1	4.1	6.8	4.9	5.5	5.11	5.97
1	3.6	2.5	3.7	2.8	4.0	1.8	3.2	1.6	2.9	2.53	3.16
5	3.2	1.8	0.9	3.0	1.5	2.8	0.7	1.2	1.8	1.57	2.08
10	1.1	---	---	---	1.1	1.1	0.6	0.4	0.9	0.68	1.14
15	0.1	---	---	1.5	2.3	1.9	2.0	1.5	1.5	1.25	1.78
**2010**											
0	7.1	11.2	7.1	9.0	12.6	12.8	11.4	7.5	9.8	9.34	10.37
1	4.9	7.0	6.1	8.4	6.7	4.7	9.2	5.4	6.6	6.13	6.98
5	0.9	3.1	4.2	3.6	---	2.1	1.8	1.4	2.9	2.69	3.20
10	0.7	1.1	1.7	2.5	2.4	0.9	0.5	0.7	1.3	1.14	1.54
15	0.5	0.4	0.6	0.6	1.2	0.5	1.3	1.3	0.8	0.65	0.96
**2011**											
0	8.7	8.6	15.1	14.4	12.8	9.0	15.7	8.3	11.6	11.05	12.16
1	10.7	4.6	11.4	12.0	13.7	7.7	7.5	8.7	9.5	9.02	10.04
5	9.7	7.2	6.4	8.4	6.0	4.0	8.0	7.7	7.2	6.74	7.64
10	9.2	6.3	5.5	5.3	5.9	11.0	7.0	8.4	7.3	6.87	7.78
15	7.4	10.1	10.2	10.7	10.1	7.3	7.6	8.6	8.9	8.35	9.34

95% CI: 95% confidence interval for the average cross-pollination rate at each distance.

The lowest cross-pollination rates occurred in 2009, with an average of 5.5% in the plants adjacent to the pollen source (Table 2). These percentages decreased significantly to 2.9% across directions (1.6 to 4.0) at 1 m, and remained about 1.5% (0.1 to 2.3) at 15 m, the maximum distance of that year. In 2010, the PMGF rates were 9.8% (7.1 to 12.8) at 0 m and 6.5% (4.7 to 9.2) at 1 m. At 5 m, the values were similar to those of 2009. The highest values at all distances were obtained in 2011: 11.6% (8.3 to 15.7) at 0 m, 9.5% (4.6 to 13.7) at 1 m, and 8.9% (7.3 to 10.7) at 15 m distance. In 2010 and 2011, additional receptors were placed in the wind direction (C and D) at distances of 20 and 25 m (it was not possible to cover all distances due to space constraints). In side C, 1.8% and 1.2% rates were recorded in 2010 at 20 m and 25 m distance respectively, while these values were higher in 2011 with 5.8%, and 4.6%. At the same distances, 1.6% and 1.4% in 2010 and 8.2%, and 3.5% rates were obtained in side D.

**Fig 2:**
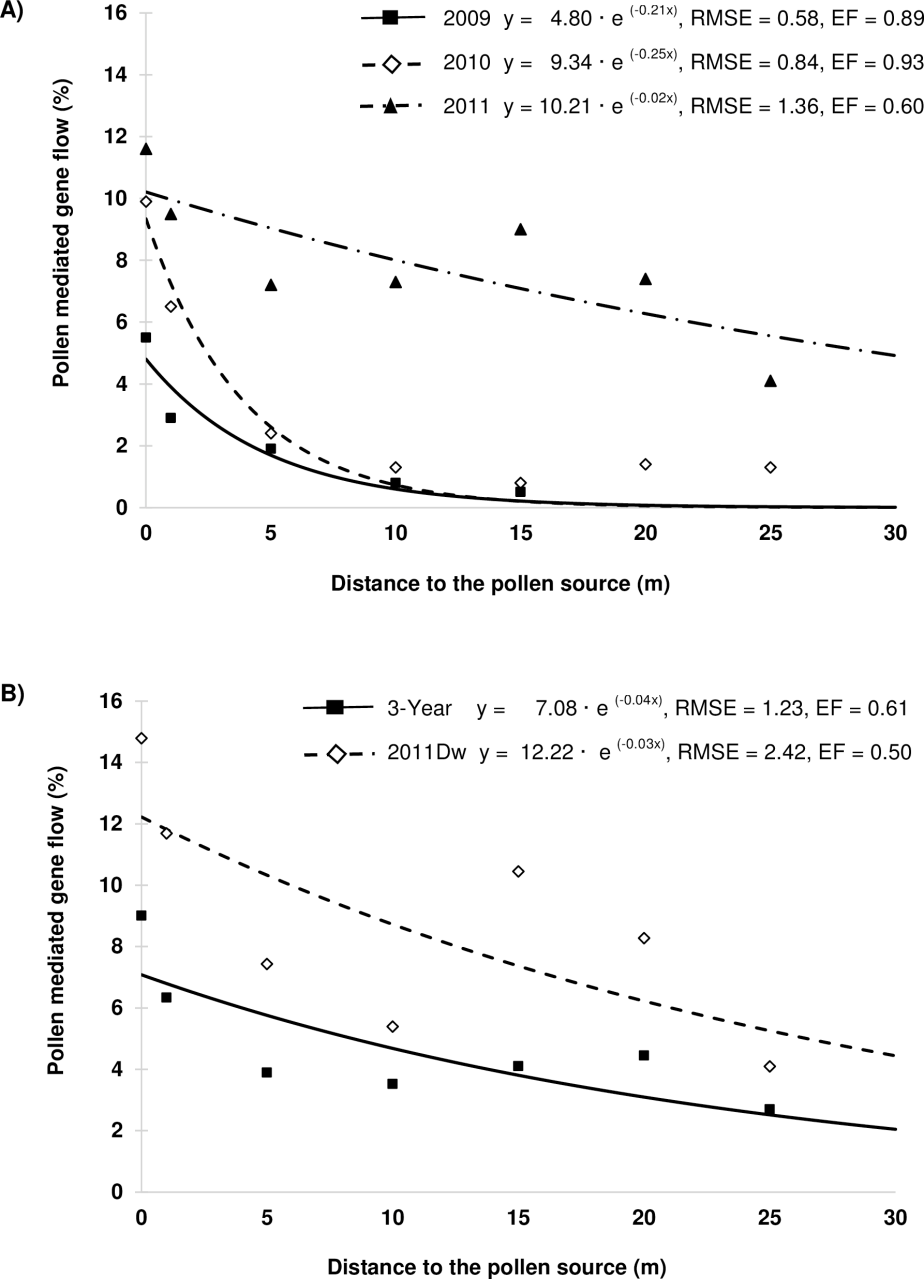
Estimated regression curves for the pollen-mediated gene flow in *L*. *rigidum* as a function of the distance. A) Regression curves for each growing season, B) Regression curves conducted with the pooled data for all growing seasons and for the worst-case scenario, with data set obtained from the downwind direction (Dw) in 2011 in which the pollen mediated gene flow was the highest.

Fig 2 shows the curves obtained by fitting an exponential decay model to the cross-pollination rates as a function of distance. Regression analyses were conducted for each year with data pooled across directions from the pollen source (Fig 2A). The RMSE values for cross-pollination among years ranged from 0.58 to 1.36 and EF values from 0.60 to 0.93 and showed the goodness of fit for the predicted models. Fig 2B shows the regression curves for the 3-year average, with data pooled across years, and for the worst case scenario with data only from downwind directions in 2011 in which the greatest cross-pollination rates occurred. For the 3-year mean and for the worst case scenario, RMSE values were higher (1.23 and 2.42, respectively) and EF lower (0.61 and 0.50, respectively), which could be due to the higher variations observed. Across years, the predicted gene flow was 7.1% at 0 m distance and declined with increasing distance to 5.7% at 5 m, 3.8% at 15 m and 2.5% at 25 m, the maximum distance of the experiment. The predicted distance wherein cross-pollination (outcrossing) decreased by 50% (O_50_) was 16.7 m. Gene flow was much higher downwind, with values of 12.2% at 0 m, 10.3% at 5 m, 7.4% at 15 m and 5.2% at 25 m. In this case, O_50_ was predicted at 30 m from the pollen source.

## Discussion

Our study provides a data set on cross-pollination rates among *L*. *rigidum* biotypes under greenhouse and semi-arid field conditions. Under open greenhouse conditions, there were great differences between the two years of experiment, with average cross-pollination rates that were three times higher in 2008 than in 2007 (44% vs 14%). These differences could be attributed to environmental conditions. Wilkins and Thorogood [36] reported that high temperatures (34°C) during anthesis increased the degree of selfing in *L*. *perenne* from 2 to 30%, thus decreasing the cross-pollination ability. This may have occurred in our study in 2007, in which mean temperatures were over 30°C for several days. In contrast, during the 2008 crossing period temperatures were lower (monthly average of 13.9°C, with a maximum of 23°C) and RH was higher than in 2007 (70% vs 56%), conditions that allowed a longer pollen viability and therefore higher rates of cross-pollination [37,38].

The environmental conditions discussed above also influenced cross-pollination in the field study. The lowest amount of cross pollination occurred in 2009, which was extremely hot and dry. These conditions probably reduced pollen viability and the pollination period, which was the shortest of the 3-year period (16 days). The highest amount of cross pollination occurred in 2011, when temperature and humidity conditions were favourable for cross-pollination. Cross-pollination in adjacent distances to the pollen source varied from 2.3 to 15.7%. As the distance between the receptor plants and the pollen source increased, cross-pollination rates decreased. This was because pollen concentrations rapidly decline at increasing distances [21,38]. At 15 m, PMGF ranged from 0.1 to 10.7%, depending on direction and year. At 25 m downwind, the maximum distance of the trial with empirical data, we recorded cross-pollination of 4.6%. Busi et al. [24] under Western Australian conditions and using sulfometuron-resistant and susceptible *L*. *rigidum* biotypes, found 12.9% cross-pollination at 0 m and 0.96% at 100 m from the pollen source. At the same distances, and using the model for the 3-year data, a 7.1% cross-pollination was estimated at 0 m. Assuming that predictions beyond the empirical data set require extrapolations whose accuracy may be difficult to determine, a 0.11% cross-pollination rate (1 diclofop-methyl resistant hybrid in 1000 S seeds) would be predicted at 100 m under our conditions. At this long distance, a rate of 0.42% would be predicted in 2011. It is important to consider the relative sizes of donor and receptor populations when considering the risks posed by gene flow. If the recipient population is very small, i.e. one or two *L*. *rigidum* plants in the field, the absence of extensive pollen competition will result in higher resistance frequencies. While Busi et al [24] used a pollen source with 1 to 10 R *L*. *rigidum* plants m^-2^ and a pair of S receptor plants per distance, in our study 100 R plants m^-2^ as pollen source and 12 S receptor plants per container and replication were used, which may have resulted in higher pollen competition. The discontinuous experimental design used, with recipient plants in patches, simulates real field conditions with patches of weeds scattered over the field at different distances from a potential source of resistance. Furthermore, weather (temperature, rainfall, air humidity, and wind speed and direction) and topography are major factors that will affect cross-pollination rates.

The distribution of herbicide resistance alleles can appear at random, suggesting multiple, independent appearances of the mutations or by pollen mediated gene flow from a single individual population or area [6,39]. This gene flow increases the initial frequency of herbicide resistance alleles in unselected *L*. *rigidum* populations [14,40]. The transfer of a single resistance allele might be enough to introduce the character of resistance in a previously susceptible population [41]. Although pollen mediated gene flow rates are dependent on the distance of the receptor plants from the resistant source, these rates are generally higher than the initial frequency of herbicide-resistant individuals in weed populations, which is generally assumed to be about 10^−6^ [42], so herbicide resistance transfer by pollen movement could play a major role in the speed at which herbicide resistance evolves in a population. Once resistance has reached a high frequency in a given population, gene flow can propagate it to neighbouring populations promoting the genetic homogeneity of the populations at the landscape level [14, 40]. In a previously conducted survey of herbicide resistance in *L*. *rigidum* populations, a significant positive spatial autocorrelation was found for herbicide response between populations located within 15 km from a highly resistant ryegrass population, evidencing a resistance hotspot due to the exposure of the populations to similar herbicide regimes in the past and distances close enough for gene flow to occur [8].

The simulation models on herbicide resistance dynamics in weed populations, reviewed by Renton et al [43], require a lot of plant-specific data. Therefore, it is necessary to generate a detailed data set for each relevant weed species before a management tools for a specific weed species and a particular herbicide can be developed. The experimental design used in this study was not large enough, due to space constraints, to allow observations of cross-pollination at long distances. However, it provides valuable information for assessing the spread of herbicide resistance genes via pollen at medium distances in *L*. *rigidum*. We fully agree with Busi et al [24] in that simulation models give no or little attention to the pollen-mediated gene flow of resistance genes related to distance and its subsequent dispersion, so its real importance could be underestimated. It is important to stress the lack of data on PMGF in *L*. *rigidum* and that the results of limited experimental data from one area may not represent the prevailing situation in another location and climate. Therefore, it will be necessary to extend these experiments on a reduced scale with more studies with larger pollen sources and longer distances. PMGF may not only play a role in gene movement between fields to accelerate resistance evolution, but it could also favor the development of multiple-resistant populations through the stacking of different resistance genes (occurring faster than resistance to two herbicides evolving sequentially), which will make it even more difficult to control resistant populations.

Herbicide susceptibility in one field will be threatened by the presence of an herbicide resistant trait in the neighboring fields. In the context of the management and mitigation of herbicide resistance, it will be important to eliminate resistant ryegrass before flowering, rather than before seed production, and to introduce integrated weed management practices with cultural, mechanical, and alternative chemical methods to reduce the potential for development of additional resistance problems. Even so, it will not be an easy task in wind-pollinated weeds, like *L*. *rigidum*, in which resistance traits can persist in individuals growing in the field margins, which can survive a multi-year herbicide mode of action rotation and re-introduce the resistance trait into the field. Other options must be studied, such as the limitation of the pollen flow by using barrier crops or zones and spatial planning of land use taking into account the dominant winds. The spatial scales are very important for resistance management, which should consider more than just the single herbicide-resistant population. Therefore, collaborative efforts of many farmers will be necessary in order to minimize occurrence of herbicide resistance.

## Supporting Information

S1 Appendix Pollen mediated gene flow in *Lolium rigidum* dataset. (XLSX)
